# Engaged Medical Humanities—A Framework for Knowledge Production in Research Collaborations

**DOI:** 10.1007/s10912-025-09976-z

**Published:** 2025-08-14

**Authors:** Louise Folker, Anna Lyngdal Wulff, Stig Bo Andersen, Line Steen Bygballe, Marie Gorm Aabo, Barbara Egilstrøð, Aske Juul Lassen, Astrid Pernille Jespersen

**Affiliations:** 1 Copenhagen Centre for Health Research in the Humanities, Saxo Institute, Faculty of Humanities, University of Copenhagen, Copenhagen, Denmark; 2 Department of Odontology, Faculty of Health and Medical Sciences, University of Copenhagen, Copenhagen, Denmark

**Keywords:** Engaged medical humanities, Critical medical humanities, Engaged research, Trading zone, Accountability, Denmark

## Abstract

In the field of medical humanities, there has been a general call to understand biocultural entanglements and to break down rigid distinctions between culture and health, as well as disciplinary boundaries. In line with this call, we suggest a medical humanities approach that further breaks down the distinction between basic and applied research. We conceptualize this approach as *engaged medical humanities*, as it both contributes to ongoing theoretical discussions and engages in empirical settings. Based on our ten-year practice of conducting medical humanities research at the Copenhagen Centre for Health Research in the Humanities (CoRe), and by zooming in on two collaborative research projects, we discuss how we realize and navigate these diverse engagements. On this basis, we offer a framework for conducting engaged medical humanities research to encourage and inspire future projects. First, we discuss how we take our main inspirations from the critical medical humanities and engaged research. Second, we identify several challenges that arise when our research approach is put into practice. We focus on our collaborations with stakeholders outside of academia, discussing how we navigate collaborative complexities while emphasizing the importance of empirical sensitivity and fluid accountabilities in studying health issues. Third, we discuss our knowledge production, encompassing various formats spanning both theoretical and conceptual contributions within academia, as well as practical interventions and instrumentation aimed at societal needs. Finally, we offer reflections on the potential we see in our framework and discuss the conditions for further developing the engaged medical humanities in a Scandinavian context.

## Introduction

The field of medical humanities is sprawling and open-ended, with threads into diverse disciplines and their theories, methods, and areas of interest. This article outlines how medical humanities research is conducted in a Danish context, specifically at the Copenhagen Centre for Health Research in the Humanities (CoRe).^1^ The present authors are all involved in CoRe, and by writing this article together, our aim is to collectively explore and share the various conditions and opportunities we have experienced working in the medical humanities. CoRe’s research focuses on ethnographic and historical studies conducted closely with stakeholders such as patients, healthcare practitioners, patients’ relatives, organizations, and authorities. Our studies of illness and health in everyday practices are engaged in a field in which actors, interests, and processes meet and diverge and in which political, social, and economic conditions mix with daily routines, expectations, hopes, and hopelessness (Jespersen 2021). With our research, we aim to uncover and discuss cultural and social complexities and challenges of health, illness, and medicine, offering insights to address them in twenty-first-century welfare states.

On this basis, we emphasize the necessity of an engaged medical humanities approach, offering this article as a framework for the *engaged medical humanities*. The framework fuses the hallmarks of the *critical medical humanities*, emphasizing the societal complexities and entanglements of health and illness issues, and *engaged research*, which emphasizes the active engagement and involvement of multiple stakeholders in solving complex societal challenges. We will state and demonstrate that this research approach transcends rigid distinctions between basic and applied research (Santos et al. 2022). We contend that prioritizing engagement with stakeholders and societal issues enriches our theoretical insights and research quality, while, conversely, our theoretical insights and contributions inform and enhance our engagement with practice, other disciplines, and societal partners. We therefore aim to discuss the potentials we see and experience in our engaged medical humanities approach, including the challenges, dilemmas, and research ethics raised by conducting medical humanities research in this manner.

With this article, we contribute a framework for thinking about engaged medical humanities encounters. It challenges the graphically neat and polished project plans we often meet in the literature on collaborative projects and in which our own and many others’ funding applications and project descriptions are presented. As we have never experienced a project following an exact plan without challenges, surprises, and changes, the proposed framework allows for reflection on the troubles and messiness occurring along engaged medical humanities project trajectories and beyond. On this basis, we seek to contribute a more realistic framework in which to think about project complexities that cannot be simplified in accurate graphical setups or anticipated in initial project plans.

With this framework, we explore how to address the troubles of the engaged medical humanities. *We emphasize that this framework is for thinking: it encourages methodological and ethical reflections, including critical and sensitive thoughtfulness about the complexities that diverse research engagements and knowledge productions bring*. Drawing inspiration from Donna Haraway’s (2016) concepts of *sympoesis* and *making-with*, our engaged medical humanities approach embodies an ethos of continual interaction and collaboration. Further inspired by Haraway (2016), we suggest that a way of “staying with the trouble” of the engaged medical humanities is to be mindful and explore how to navigate the complexities of various research engagements, which is exactly what this framework allows.

As our research engagements are always socio-materially situated, the framework necessarily takes shape in the particular research projects’ settings and in the situations explored. We therefore introduce the engaged medical humanities via specific insights from two research projects to provide inspiration and guidance for critically reflecting on the challenges inherent in engaged medical humanities endeavors. The framework described here is thus formed by our analysis and discussions of these projects to illustrate and generate knowledge of how the messiness of the engaged medical humanities can be navigated. We present two sets of recurring concepts that have regularly surfaced across our research endeavors over the past decade. These concepts represent a selection of collaborative and academic challenges, obstacles, and potentials that are at stake in different ways. First, through the concepts of *trading zone* and *accountability*, we examine how mutual commitments and obligations in collaborative projects can both facilitate and impede research and interventions. Second, through the concepts of *theorizing* and *instrumentation*, we address the balance and entanglement between theoretical insights and practical applicability. By introducing this framework, we invite colleagues to utilize it to think critically about and address the chaos that diverse research engagements bring.

## State of the art

The critical medical humanities have called for new reflection on how the humanities can critically engage with, relate to, and collaborate with the natural sciences, biomedicine, and health professionals (Carusi 2016; Engebretsen et al. 2020; Hansson and Irwin 2020; Macnaughton 2011; Viney et al. 2015). In opposition to the medical humanities, as the “supportive friend” of the medical sciences (as proposed by Brody 2011), which for decades has investigated health and medicine from varying humanistic perspectives, Viney et al. (2015) notably argued that taking a critical stance involves a more intensive engagement with “how health, illness, and treatment are constituted in and through tangled webs of human and non-human biosocial organisms, political–economic formations, discourses and affects” (2). Calling for both involvement in and the disruption of dormant “biomedicine truths” (Macnaughton 2011), critical medical humanities scholars insist on an inherent cross-disciplinary entanglement with the knowledge production and practices of biomedicine and the natural sciences (Fitzgerald and Callard 2016). In other words, they urge us to collaborate, engage, take risks, and intervene in health issues more directly (Engebretsen et al. 2020; Kristeva et al. 2018). We draw significant inspiration from this tradition, which forms an important cornerstone of our research approach, but as a predominantly theoretical development, we contend that the discussions taken by the critical medical humanities would benefit from empirical scrutiny (Fitzgerald and Callard 2016). Consequently, as a significant aspect of our research at CoRe, we prioritize an empirical, richly textured sensitivity (Whyte 2009) in our engaged medical humanities approach, exploring the analytical consequences of concepts such as entanglement and the bio-cultural and their practical relevance and applicability. In line with discussions of critical proximity in research (Birkbak et al. 2015), being “critical” of the critical medical humanities means that our engagements are not simply formed by a negative critique of the medical but as constructive and active engagements and involvements with health researchers, health policy, practitioners, etc. (Macnaughton 2011).

Similarly, engaged research frameworks have proclaimed that issues of health and illness must be understood as nested in an array of complex social and environmental systems, calling for projects that engage and collaborate with all key stakeholders (Barkin et al. 2013; Haapanen and Christens 2021). In recent years, this has led to several reports, how-to guides, and frameworks emerging from research teams entering engaged research projects suggesting a stringent engagement process, determining the value of engaged research and its societal impact (Barkin et al. 2013; Campus Engage 2022a, 2022b; Cunliffe and Scaratti 2017; Haapanen and Christens 2021; Heney and Poleykett 2022; Lloyd et al. 2012). While our engaged research approach at CoRe is not clearly rooted in these frameworks, their discussions help us mitigate the aforementioned call of critical medical humanities scholars to engage and collaborate more directly with a broad range of actors in the welfare sector. The engaged research guidelines outline different project stages from ideation through data collection, analysis, implementation, and anchoring, often illustrated through cogwheels and phased process models culminating in a “light bulb” moment. It is a key principle of these guides that engaged research is “advanced with societal partners rather than for them,” emphasizing the importance of an initial alignment of expectations regarding “engagement approaches,” “responsibilities,” and “roles” outlined before project kick-off (Campus Engage 2022a). Yet, while we acknowledge and recognize elements of our own processes in these recommendations, we emphasize that the project realities rarely conform to such generic frameworks, which smooth out the important work and ad hoc processes of maintaining, repairing, and negotiating the collaborations. When engaged research is depicted as mutually beneficial and based on alignment and joint agreement, it standardizes expectations and equalizes engagement since everything needs to be prearranged and predetermined. We argue that these generic frameworks, although recognized, neglect the complexity of the projects. For instance, the guidelines from Campus Engage state: “In real world applications, engaged research is messier and not as linear as depicted in the diagram shown in this document. Thus, the Engaged Research Framework, like Beck’s tube map of London, is not a perfect representation; instead, it is a simplified model designed to encourage researchers to develop a clear and comprehensible plan for who is engaged across the lifecycle of the research project—when, why and how” (Campus Engage 2022a, 3). Even though the guidelines recognize the lack of real-life representation in the plans, they still call for a “clear and comprehensive plan.” We agree that research projects need plans but maintain that the ideals of clearness and comprehensiveness promoted by the guidelines obscure the constant negotiations, balancing, and reformulation that constitute good project processes.

In line with recent discussions in the field of science and technology studies (STS), especially discussions of STS research as “Making and Doing” (Downey and Zuiderent-Jerak 2021), we diverge from engaged research frameworks and the way they accentuate and model collaboration and hence knowledge production as a somewhat linear trajectory extending from idea generation to end result and impact. As discussed by Downey and Zuiderent-Jerak (2021), such models tend to pass over the situatedness, messiness, and nonlinearity involved in knowledge production and knowledge travel. We concur with this criticism and pursue our engaged research in a more fluid, theoretical, and curiosity-driven manner than is typical of most engaged research frameworks, which is why our framework invokes unfolding project-specific experiences, conflicts, ad hoc situations, and (re)negotiations occurring during the project trajectories. Drawing inspiration from the critical medical humanities, the engaged research framework, and STS, our ambition is to devise an engaged medical humanities with an empirical, socio-material and (everyday) practice-based approach, which seeks accountable engagements inside and outside academia.

## Two engaged medical humanities projects

In our analysis, we focus on two specific collaborative projects conducted at CoRe: *Lifelong Oral Health* and *Senior Practice*. These projects serve as examples of our research approach, acting as conduits between academia and broader societal stakeholders, as well as between traditional conceptions of basic and applied research. One project is based on engagement with healthcare institutions at a municipal level, whereas the other is based on engagements with a funding body and private companies. Our projects disseminate insights and initiatives widely, impacting not only the specific communities under investigation but also diverse research domains. Consequently, we investigate how this dynamic unfolds in practice—first, by describing the projects.

### Project Description: Lifelong Oral Health

Lifelong Oral Health is a combined research and co-creation project initiated in response to a societal challenge: the escalating prevalence of dental issues and oral diseases among the aging population, particularly older persons in vulnerable life circumstances requiring professional care (Rouxel et al. 2017). The repercussions are profound, encompassing weight loss, malnutrition, and significantly heightened susceptibility to infectious diseases when physical or cognitive impairment impedes dental self-care (Klotz et al. 2018). The overarching project questions are as follows: Why is oral health poor among older people in vulnerable life situations? What initiatives can improve it? The project has unfolded over a five-year trajectory, extending from 2021 to 2025. Our closest project partners are dentists from the Community Dentistry research group at the Department of Odontology at the University of Copenhagen. Together, we work with two Danish municipalities—Greve and Frederiksberg—which are both collaborators in and focal points of our data collection, fieldwork, and testing of co-created initiatives. We work with local project teams set up at five different care units in the municipalities, encompassing homecare units, nursing homes, and a rehabilitation center. The teams comprise diverse stakeholders, ranging from healthcare workers and dental professionals to project managers, local care unit managers, and town hall managers. The research design encompasses register-based studies, questionnaires, clinical registrations, and—CoRe’s primary area of responsibility—ethnographic field studies consisting of semi-structured interviews with older persons, relatives, care workers, dental practitioners, and various managers, along with focus group interviews with diverse municipal stakeholder groups. Furthermore, we have conducted participant observations and facilitated co-creation activities with all partners.

#### Project description: Senior Practice

Senior Practice^3^ is a research project aiming to generate knowledge of mental well-being among older workers at small and medium-sized companies, and to find ways to promote it. The project focus comes from a significant challenge in the Danish labor market, where there is a shortage of labor and a demand for new initiatives to extend working life. Hitherto, political responses to extending working lives have focused on retirement reforms, economic incentives, and so-called “senior days” (monthly or weekly days off). A less prominent focus in the response has been on understanding the quality of later working life, including mental well-being, workplace dynamics, and the proper use of experienced workers. The overarching questions of the project are: What constitutes good later working lives and retirement transitions among older workers in a Danish context? How can companies support older workers’ mental well-being? The collected data consist of semi-structured interviews with older workers and their managers, union representatives, and/or human resource (HR) personnel, combined with participant observations with older workers to generate insights into their work practices and dynamics with colleagues and managers. The project is funded by the Velliv Association, which, according to its guidelines, at the time of application, did not grant funding for more than one year at a time. Therefore, the project has been conducted in three distinct phases based on three different research applications.

## Trading zones and accountability

In this section, we examine the partnerships and stakeholder relations in our two projects. Specifically, we want to explore the opportunities and challenges inherent in engaging with societal partners, including funding bodies. Drawing on the American philosopher Peter Galison’s (1999) concept of the *trading zone*, we seek to emphasize and reflect on the ontologically heterogenic exchanges that engaged research often entails. In addition, we use Donna Haraway’s (2003, 2016) expansive reflections on *accountability* to explore the dynamic, hierarchical, and ethical complexities these collaborations imply. We utilize these concepts to break away from conventional project plans, arguing that a more nuanced framework is essential to capture the various navigational issues of the engaged medical humanities we propose.

The guidelines stemming from engaged research (Barkin et al. 2013; Campus Engage 2022a,b; Cunliffe and Scaratti 2017; Haapanen and Christens 2021; Heney and Poleykett 2022; Lloyd et al. 2012) often seem to emphasize stable and reciprocal relations, the predetermined alignment of expectations, and the assumption that all parties give and receive as central to successful project management and collaboration. As such, the guidelines emphasize a social contract and a plan that determines the sequence of actions of each partner or stakeholder. However, we note that these guidelines fail to capture the asymmetry, power dynamics, temporal fluctuations, and heterogenic stakeholder interests in the collaborations. In our experience, project relations are not stable social obligations, as they change and evolve throughout the projects and beyond. They are prone to sudden shifts in engagements and relations. The changing project contexts invariably shape trajectories and may hijack the pre-established agreements, or force the ongoing renegotiation of collaboration terms in unforeseen directions.

We find it useful to reflect on these complexities in terms of Galison’s (1999) concept of the *trading zone*. Galison introduced the concept to facilitate exploration of scientific exchange between schools of physics unaligned within the same Kuhnian paradigms (Galison 1999; see also Collins et al. 2007), comparing this exchange to classical anthropological studies of trade between groups without a shared language. To Galison (1999), the physicists represented different subcultures that did not speak the same scientific language: “Like two cultures, distinct but living near enough to trade, they can share some activities while diverging on many others. … They can come to a consensus about the procedure of exchange, about the mechanisms to determine when the goods are ‘equal’ to one another” (p. 146). The trading zone is a site of exchange that is locally coordinated and mutual among heterogeneous actors without conflating the differences and disunity of the involved communities. According to Galison (1999), these subcultures are enacted through different means, such as goods and bargains, and by developing a pidgin language with which to communicate through exchanges, which do not necessarily approach ontological agreement but recognize mutual benefit (Elgaard Jensen 2020). As emphasized by Collins et al. (2007), the notion of the trading zone concerns problems of communication and coordination that require the handling of differences and heterogeneity as the participating sub-cultures are not necessarily on an equal footing. Crucial for our analysis, these actors do not evaluate the exchanged goods uniformly, as the elements of exchange come with different degrees of importance or symbolic, cultural, or other kinds of value. Working as “social and intellectual mortar,” a trading zone temporarily binds together communities and coordinates actions and beliefs despite ontological differences (Galison 1999). Understanding engaged research projects as trading zones, the intercultural bargains made across the ontological, contextual, sectoral, and administrative differences that inform the differentiating valuations of exchange become key in understanding and bolstering the continual negotiations and collaborations. As an alternative to the neatness of the engaged research guidelines, we find inspiration in this concept because it allows us to think about and address the heterogeneous and dynamic nature of the engaged medical humanities. In addition, it provides an opportunity to reflect on the various considerations and obligations—whether organizational, political, or personal—that we and our project partners face.

It is central to our argument that entering such trading zones makes us as researchers accountable to all involved project actors and stakeholders in ways that transcend mere adherence to predetermined contractual obligations. Our accountabilities extend to diverse stakeholders who contribute to and are impacted by the projects and evolve in ever more complex and negotiated pathways throughout the projects. In our collaborative projects, the stakeholders encompass the research community, funding bodies, (project) managers, employees, and individuals, including patients and their families. Each group has a stake in the project outcomes, although with varying degrees of formal recognition or mutual commitment. In exploring accountability in these complex collaborative environments, we find resonance in Donna Haraway’s (2016) expansive concept of accountability, understood as an active, situated practice rooted in relationality rather than any universal or abstract moral obligation. Haraway’s (2016) understanding of accountability emphasizes dynamic and asymmetric connections and engagements, as “we are not all response-able in the same ways. The differences matter – in ecologies, economies, species, lives” (29). For Haraway, accountability is not simply about delivering solutions but also about ongoing, mutual learning and adaptation occurring when multi-actor networks collaborate to create flourishing future conditions. Haraway has highlighted the complex and conflicting nature of collaborative processes, noting that it “makes strong demands on the littermates” (2016, 110) and requires “understanding how things work, who is in the action, what might be possible, and how worldly actors might somehow be accountable to and love each other less violently” (2003, 7), even in the face of “barely possible but absolutely necessary joint futures” (2003, 7). Haraway’s concept of accountability further involves recognizing and addressing power dynamics in these collaborations to counteract dominance and control. Inspired by Haraway, we therefore acknowledge both the formal and informal hierarchies in our own and the collaborating partners’ organizations. We thus draw on Haraway’s concept of accountability in our engaged medical humanities framework, encompassing both our ongoing methodological considerations as well as the constant ethical and navigational reflections that guide our diverse engagements. This entails actively engaging with the needs and concerns of all stakeholders while also recognizing that we cannot accommodate every perspective. We thus move beyond static notions of accountability toward a more fluid and adaptive approach to work for more inclusive forms of engagement, ultimately enhancing the integrity and impact of our research outcomes. Together, the concepts of trading zone and accountability underscore the importance of recognizing and navigating the difficulties inherent in engaged research collaborations. In particular, this combination of concepts facilitates an understanding of the power imbalances, unevenness, and asymmetries within the project trading zones and across the various (trading) partners involved.

### Case: Senior Practice

It is a significant part of the Velliv Association’s aim to support projects that enhance mental health across work life stages and to ensure that any insights gained are translated easily and quickly into tangible, practice-oriented tools that benefit the Association’s target group of small and medium-sized companies (SMCs). According to the Velliv Association, SMCs seldom have the means and resources to ensure good mental support infrastructures for their workers; accordingly, the Association sees it as its mission to connect research on work life and mental health with practice through interventions. For researchers, this means that their projects must address challenges and needs experienced by SMCs, that SMCs must be involved throughout the projects, and that the results must be applicable in practice. For the Velliv Association, these requirements may seem logical and justified, but, as we will describe, they refer the responsibility to create engaged collaboration to the researchers, thereby installing an inherent asymmetry in the project relations.

During our initial preparations for the research project, the Velliv Association requested that a specific number of companies of a specific size be recruited for the project. To meet these requirements, we depend on companies being willing to participate so that we can pursue our research interest in investigating the mental well-being of older workers. Reaching out to, communicating with, and finally recruiting companies, however, involve a meticulous effort to align the specific interests of the companies with the overall purpose defined by the Velliv Association and our research interests. Local managers have to approve their employees using working hours to participate in the research project, taking time away from essential work duties, while estimating that this time will, in the long run, be profitable. Sometimes, unforeseen situations change that calculation, so we have experienced some companies withdrawing from the project due to shifting resources and priorities.

Two benefits we can offer the companies in exchange are our research expertise as well as our capabilities in developing practice-oriented instruments that could potentially benefit their work environments in the future. While the companies acknowledge the relevance of the research project and its aims and concur with the importance of the project aim, they may still find it challenging to determine whether the time investment is worthwhile, especially due to their small size, lack of resources, and uncertainty about the added value of our tools. The challenge we face, and the responsibility we have been given by the Velliv Association, is to convince the companies that, together, we will be able to address and develop solutions to problems that are close to them. One way of understanding our challenge is how convincing we must be in outlining the contours of a promising trading zone that resonates with the companies’ interests and strategies. To be able to commencing a project depends on creating a joint but localized zone of activities, enabling coordination and exchange among the disparate actors involved. Furthermore, the objects, bargains, and pidgin language shared in the trading zone may serve as enablers of the activities (Galison 1999; Elgaard Jensen 2020).

In the Senior Practice project, we have observed that some companies are motivated to participate by the opportunity to brand themselves as socially responsible by engaging in a research project that addresses a high-profile societal issue, namely, the mental health and retention of older workers. For example, HR personnel advocated participation by emphasizing its positive significance and good publicity for the company. They saw collaboration with the university as a stamp of approval of their policies for older workers and as a way to boost their image as socially responsible companies. Photographs showing the researchers with company representatives became objects with different cultural and symbolic values, sometimes resulting in awkward trading exchanges. The companies featured the photographs in their newsletters to promote their image; similarly, we used the photographs in research communication to document our empirical and strategic engagements.

The project is closely linked to the current debate on ageism in the Danish labor market. The ageism debate is a “hot topic” in many sectors and among many actors of the labor market, often creating stark and contrasting positions and conflicts that could influence the collaboration. Partly to recognize and address this debate and partly to avoid possible severe disagreements between the participating partners (researchers as well as companies) regarding the interpretation and accountability for ageist practices, the project has developed a common language with which to mitigate any differences—a sort of pidgin language. Key to this pidgin language has been renaming the project in several communication outlets. It became especially clear to us that the term “senior employee” has negative connotations of being worn out and ready for retirement. Therefore, we have changed the project name used in the project’s website, reports, and dissemination activities (e.g., a podcast) to The Experienced. These apparently insignificant situations and consensus-seeking activities, which can be understood as bargaining outcomes in trading zone terms, have a huge impact in creating a sense of accountability and connectedness—a sense that, in both cases, is not clearly described in the initial project description.

The Senior Practice project exemplifies how current Danish funding conditions call for complicated engagement among very different actors, with different mindsets, working practices, and ways of understanding the added value of collaboration. The case also highlights how the requirements of the funding landscape often refer the responsibility for creating and facilitating collaboration to the researchers. This responsibility is time-consuming and precarious to discharge, involving unforeseen bargaining situations. Applying the notions of the trading zone and accountability to understand these situations highlights the unevenness and asymmetry involved in collaboration. However, doing so may also bolster researchers’ efforts to create relevant incentives that project partners find attractive and through which they can become connected to and accountable for the project. Altogether, the trading is often both awkward and difficult and requires ongoing thoughtful consideration. Yet, we consider it an inevitable precondition for many engaged medical humanities projects in order to gain access to and position us as researchers in strategically important arenas. It thus gives us a unique strategic opportunity, for example, to disseminate humanistic knowledge production in specific and deliberate parts of society.

### Case: Lifelong Oral Health

In Lifelong Oral Health, we engage in various arenas and discussions inside and outside academia. Through our multifaceted engagement and exchange among diverse actors and fields, we navigate a web of entanglements. We are accountable in multiple directions. In the Lifelong Oral Health trading zone, we are bound by obligations to our project collaborators, encompassing contractual agreements, joint applications, ethical considerations regarding informants, and legal framework commitments. Simultaneously, we are accountable for our freedom of inquiry and autonomy. Accountability in the Lifelong Oral Health project is therefore not an immutable principle; rather, it is susceptible to challenges and fluctuations. It is never fixed, meaning that our accountabilities to all the various actors, including ourselves, can collide with one another. In addition to being responsible for ethnographic studies, our work requires constant and sensitive navigation in a highly interdisciplinary and intersectional project design.

On one occasion in particular, our different accountabilities gave rise to conflict that, for a while, destabilized the collaboration with one of the municipalities. In this case, we can use the trading zone and accountability concepts to think about a specific conflict situation that suddenly arose during the project. The conflict centered on criticism of our municipal partners and their management of dental care, criticism voiced by the relatives of older persons in nursing homes whom we had interviewed in the project, and whether this criticism should be mentioned in a national report we were to publish about the insights and initiatives of the Lifelong Oral Health project. During our fieldwork, the dismayed relatives often described inadequate dental care as an example of how they believed their loved ones were not being adequately treated within the elderly care system. For instance, they said: “You have to get so ill before you get help here. Dental care is a good example of how the basics don’t work in this nursing home”; “I actually thought the system would step up for us. I’m so hurt and angry about it. Excuse me, but should we try the welfare thing or what?”; and “It’s a declining nursing home.” Their criticism is relevant to the project since relatives often play a role in procuring dental care supplies, registering and scheduling appointments with dentists, and providing significant support to the care workers when older persons are confronted with dental issues and need professional daily dental care.

In the context of drafting the project report, we informed the municipal stakeholders in the project’s steering group that the relatives’ criticism would be mentioned in the report. Immediately afterward, the project managers and local managers in one of the municipalities emphasized that this worried them, and they asked us to ignore the criticism, as they did not find it relevant to the project or fair to the care workers. Additionally, they asked us for editing rights regarding the report and future communications and to be more loyal to the project. Although they did not explicitly threaten to withdraw from the project, they said things like “We didn’t sign up for this,” expressing doubts about their participation. At first, the weight of their concerns and requests took us by surprise. At the same time, we were alarmed about their request for editing rights. The project collaboration was immediately threatened as this conflict exposed the diverging accountabilities between two organizational “subcultures”: on one hand, the municipalities risked political consequences and workplace repercussions because of our publishing of harsh criticism; on the other hand, we as researchers risked our research freedom and integrity if the criticism were not published.

The conflict lasted a few days, during which communication and attempts at mutual understanding were intensified. We initiated careful dialogue while ensuring that the communication was routed through one researcher and one municipal project manager, who had previously had good collaboration. They had several telephone calls characterized by personal trust and affective competence. During these calls, the municipal project manager referred to a huge breaking news scandal in the media about failures in Danish elderly care, which was notable and concurrent. It became clear to us that our partners were concerned that the quotations of the relatives would seep into the public debate, putting our partners at risk of a media scandal. In the phone calls, we clarified that our focus in the report would be on recommendations around the relatives and that the criticism would not be framed sensationally, while emphasizing our freedom of research and editing rights over our publications. The project manager quickly became comfortable with the situation. The next few days of close communication therefore concerned making the other actors in the municipality—managers of different care units and upper-level managers at the town hall—feel comfortable. We formulated a reassuring email in careful, soothing, and de-escalating pidgin language, expressing our understanding of their situation while also presenting the importance of our freedom of inquiry, including not sharing editing rights. In addition, a joint meeting was arranged to address the issue; however, when all municipal actors responded to the email, saying that they were comfortable with the situation and that they had not considered the problematic nature of wanting editing rights and waived their demand for them, the meeting was cancelled.

Nevertheless, the conflict made it clear that the trading zone of this engaged research project was formed by the fact that in a universal, public healthcare system like Denmark’s, elderly care is the task of municipalities, which are politically governed organizations subject to elections every four years. As a result, municipal leaders face considerable scrutiny from the media and fear of potential scandals. This makes engaged research with municipalities a difficult terrain to navigate, as, in this case, our accountabilities to the municipal stakeholders, the relatives, and ourselves as autonomous researchers collided. The situation created dilemmas, as we were aware of our formal and ethical accountabilities to everyone involved in the project, including ourselves, while being deeply interested in maintaining good stakeholder relations and securing the project. By understanding this conflict in terms of the concepts of trading zone and accountability, we stressed the project’s complexities, which could not have been entirely predicted or prevented by institutionalizing the reciprocal relations and commitments through formal mechanisms such as legal instruments, contractual agreements, and oversight by ethical review boards. At the moment of potential crisis, our primary navigational device was not to return to the contract and the plan but rather to engage with the project as a trading zone: a place and a specific opportunity for encountering specific people and specific objects that could be brought together—opportunistically and pragmatically—on this occasion to solve the problem at hand. We also leaned into Haraway’s (2016) idea of “staying with the trouble” and navigated the conflict through personal trust, pre-existing relationships, and affectual competence in order to discharge our various accountabilities. To us, this case exemplifies how engaged medical humanities projects are always situated and affected by local hierarchies, individuals, work environments, and, in this case, public and political atmospheres.

Viewing the various exchanges involved in engaged research through the conceptual lenses of the trading zone and accountability allows us to acknowledge that, while we and our partners may agree on the value and mutual benefits of these exchanges, we often approach one another as ontologically distinct “subcultures.” We argue that the “trouble” inherent in these interactions is not only unavoidable but also productive. It is exactly through this trouble that we navigate and conduct the collaborative work of engaged research. Following Haraway’s (2016) emphasis on “staying with the trouble,” we seek to embrace the complexities and challenges encountered. For us, these concepts offer valuable analytical and practical guidance for understanding and addressing these challenges in project-specific contexts, regardless of whether the partners are municipalities, companies, or entirely different collaborators.

## Theorizing and instrumentation

The practical outcomes of the research process are an overarching focus of engaged research (Mikesell et al. 2013). For the same reason, engaged research is often criticized for being overly simplistic and atheoretical (Delgado et al. 2011) and sometimes even in opposition to and hampering academic practice (Paphitis 2017). Simultaneously, there has been an increased demand for research that is relevant to a broader audience and has the potential to develop new knowledge, services, and/or technologies that can have practical implications. The appeal for societally relevant research is also reflected in the requirements of the current funding landscape, which often rewards product-oriented projects (Hinchcliffe et al. 2018). This often leads researchers to attune their research ideas to applicability, which feeds into discussions of the instrumentalism of privately funded research, such as critical discussions of the expansion of neoliberal ideals into spheres not traditionally included in market logics (Oladi 2013). The ongoing discussions of applied research also point to a potential conflation between consultancy work and research, exposing applied research to criticism for losing its responsibility to be a critical reflexive voice.

Despite the very relevant concerns raised, the criticism also advances an inherent dichotomy between theorizing and instrumentation, which we find problematic. Here, we argue that combining advice from engaged research with the theoretical ambitions of the critical medical humanities will promote a research approach that holds the potential for advanced theory building in parallel with developing practice-sensitive and applicable instruments. In our experience at CoRe, we have found that the path to conducting “good” engaged research involves a dynamic critical and creative exchange between theoretical thinking and what we have chosen to call instrumental thinking. Through this interplay, our research becomes both societally and scientifically relevant.

Theorizing-cum-instrumentation is an experimental work practice in which knowledge productions (both empirical and theoretical) are conjoined to foster and cross-pollinate practical and theoretical sensibilities. A key inspiration for our experimentation is Stengers’ (2005) call to slow down reasoning. Although the project-based and thus time-bound character of our research at CoRe may seem in opposition to a call for slowing down and hesitation, by conjoining, confronting, and contrasting theory and tool making, we attempt to slow down by other means. In her cosmopolitical proposal, Stengers (2005) forefronts “the idiot” as a figure who, by slowing down and resisting conventions and consensus, produces an interstice (by asking what we are busy doing) and thereby creates a space for hesitation. The dynamic interplay between theoretical and instrumental thinking is our interstice, our site for hesitation, and our way to stop and (re)think: How can we make our knowledge production relevant and good? The tools we develop with practitioners—the so-called instrumentation of research—are embedded in theory, although sometimes invisibly. Simultaneously, it is by thinking with the practitioners and emphasizing their practices when developing tools that we build theory and materialize it. Therefore, theorizing and instrumentation are not opposites, but arguably can and should be deeply entangled when conducting engaged research. In the following, we explore the dynamic interplay between theorizing and instrumentation in the form of navigational issues that have emerged in the two projects because of different temporalities, collectives, inscriptions, and success criteria between the academic and external partners regarding the knowledge production, issues that are resolved in practice as we understand and handle it through the concept of theorizing-cum-instrumentation.

### Case: Lifelong Oral Health

In our investigation and mapping of barriers to oral health in Danish elderly care, one of our key findings is that assisted daily tooth brushing is highly shameful and intimidating for the cared-for older people as well as the care workers. We found that older persons often experience shame related to their teeth and dental issues and that care workers find it intimidating to confront these issues and the mouth as such. This insight was gained through a collaborative process melding ethnographic narratives, activities with stakeholders, and theoretical discussions. This process has led to the conceptualization of tooth shame as a unique manifestation of bodily and health-related shame and, further, to a conceptualization of intimidation in care work. On one hand, these conceptualizations developed as we presented and discussed our findings with our municipal project partners. In particular, the care workers have welcomed the findings: they describe the findings as helping them understand behaviors and atmospheres regarding dental care, enabling their work. They have embraced the concepts and further developed local initiatives and instruments to address tooth shame. For instance, they digitally register dental issues and tooth shame and use individualized pocket cards to share tips and motivating strategies among care workers for each nursing home resident in need of dental care. Furthermore, the care units have disseminated the concepts and their initiatives to address them to other care units in the municipalities that are not part of the Lifelong Oral Health project.

On the other hand, the conceptualizations are highly informed by recent academic discussions of shame and intimidation. When we began to investigate these discussions, it became clear to us that both shame and intimidation are well-documented, yet underexplored, barriers to treatments, healthcare encounters, and health as such (Dolezal and Gibson 2022). Therefore, we became interested in how shame acts as a barrier to oral health in practice, which initiated follow-up fieldwork with this specific focus. On this basis, we have published a paper on how shame acts in socio-material settings and how it can be addressed in practice (Folker et al. 2025). Most research on shame is highly focused on philosophical, psychological, and individual-based approaches to shame. However, the Lifelong Oral Health project has allowed us to investigate how shame works in a collective practice. As such, the project’s commitment to developing interventions through concrete collaborations is a key component of the novel focus on the social distribution of shame among the cared-for older persons, relatives, care workers, etc., in a collective and systemic care setting.

Our theorizing-cum-instrumentation work has sparked interest from other medical humanities research milieus, as we have been invited to present our work in lecture series abroad. This interest from international medical humanities researchers has not been related solely to teeth or oral health; instead, it has concerned our ethnographic socio-material approach to investigating shame. Furthermore, we have established international collaborations with researchers from the medical humanities to address tooth shame. It is the constant dialogue among our practice interventions, instruments, and theoretical work that creates the interstice allowing for a slowing down by other means and shapes our knowledge production. In this way, both our theoretical work and our practice interventions move within new arenas, far beyond the initial project design. The knowledge production process is manifested in both theoretical contributions and practice interventions that could never have been planned for or predicted at the project’s beginning. Moreover, the concept of tooth shame has evolved in ways in which we, as researchers, are not necessarily involved regarding both theoretical discussions and practice interventions. To sum up, our theoretical work and practice interventions not only go hand in hand but also inform, enrich, and take shape by each other.

### Case: Senior Practice

Collaborating with a private foundation such as the Velliv Association is an opportunity to have research-based impact in the field of later working life, a time characterized by many stereotypes and prejudices. One of the most significant insights achieved during the first two phases of Senior Practice from 2019 to 2022 concerned the many stereotypes and prejudices surrounding older workers. Often, managers, coworkers, union representatives, and older workers themselves would state that older workers were slow, unproductive, or unmotivated. Throughout the fieldwork period, however, we observed that such generalizing and marginalizing statements were often contrasted with the actual older workers employed at the companies. As such, a distinction was made between the specific older worker in question, who was portrayed as energetic, productive, and motivated, and “the general older worker.” Furthermore, managers and coworkers would seldom praise older workers to their faces; consequently, many of the older workers were afraid that they were perceived in line with their own prejudices concerning “declining older workers” who were “worn out.” Although confident that they were still good resources for the companies, the ongoing lack of appraisal, combined with small comments about their age from managers and coworkers, would lead to internalized ageism. This affected their well-being and self-image but was often concealed from their surroundings. In many cases, we saw this as influencing the retirement decision, which could have been prevented with timely, relevant, and caring dialogues between the manager and the older worker.

This was a phenomenon we had not encountered in the existing literature, nor had it been addressed in existing tools for practitioners. We did, however, find many similarities between this psycho–socio–cultural complex and what has been termed “the impostor syndrome” by the American psychologists Clance and Imes (1978). Hence, from our data, we crafted the theoretical concept of “the worn-out syndrome” with which to understand these age-specific challenges in later working life. While the impostor syndrome addresses the insecurities early in working life, when young people doubt their own abilities and whether they deserve their positions, the worn-out syndrome addresses the insecurities of later working life (Aabo et al. 2023). Through our analysis and the development of the worn-out syndrome as a concept, it became clear that the participating companies needed tools with which to engage in dialogues about the practices and challenges in later working life.

When applying for funding, we promised the funder that we would create practice-relevant instruments; simultaneously, we had the freedom to wait for insights from the fieldwork before deciding what such tools should consist of. During this process, and in collaboration with societal partners, such as unions, employers, associations, pension funds, municipalities, companies, and nongovernmental organizations, we developed three tools with the aim of enhancing dialogues between managers and older workers. We established these collaborations with societal partners, inviting them to engagement and discussion in workshops, informal meetings, and the like. We wished to promote the idea that recognition and appraisal are still important in later working life, and that to extend working life, companies need to involve older workers in designing their working lives. This includes flexible working hours, reduced working hours, more vacations, fewer management responsibilities, and, importantly, the recognition of existing resources, competences, and potentials to battle ageism in the company. In this process, we actively utilized the worn-out syndrome as a theoretical concept to apply when developing these tools. In other words, the worn-out syndrome has become a means for both our academic endeavors and tool development. Additionally, the tools became a means of materializing and conveying the worn-out syndrome, with the aim of anchoring research insights in practice in order to transform it and, as such, manifest its importance and impact.

One tool targeted management and HR personnel to promote the development of workplace policies to increase the focus on older workers; another tool targeted the older workers’ closest managers and was intended to facilitate later work life interviews with older workers; and a third tool aimed to prepare older workers to discuss their thoughts, during later work life interviews, about lifelong learning, gradual retirement plans, and dream scenarios. In the tool for these later work life interviews, the guide is introduced with the words: “It can be sensitive to talk about age in the workplace. But research shows that age can play a positive role in working life if you manage to create a constructive and appreciative dialogue.” Additionally, the manager is encouraged to initiate the interview by recognizing the older worker: “You are a valued employee whom I would like to keep as long as possible. We can continue as before, but if you have any wishes for us to organize the work in a different way, I am open to it. That is why I have invited you to this conversation.” Although the worn-out syndrome is never mentioned in the tools, its presence is apparent. The worn-out syndrome also became an active resource not only in our thinking but also in the use of the tools, as stakeholders seem to endorse the tools exactly because of their academic origins and the authority given to the worn-out syndrome concept through its academic use.

Utilizing the worn-out syndrome as a theoretical concept enhanced our understanding of some of the challenges at stake in practice and the factors constituting a good later working life. Subsequently, among other theoretical concepts, the worn-out syndrome has been utilized as an analytical resource in our latest academic article to analyze the dynamics regarding competence development in later working life (Wulff and Lassen 2024). We continue to explore and develop its theoretical potential in our research, making it into an instrument with which to understand the complex dynamics among older workers and their surroundings. Our fieldwork data and experimentation with tool development keep informing the worn-out syndrome as a theoretical concept, and vice versa. On this basis, working closely with a private foundation and the companies has given us access and the opportunity to investigate and intervene in critical empirical settings regarding mental health and later working life. It is the theorizing-cum-instrumentation work that makes worthwhile all the troubles of the project trading zone, as described in the Senior Practice case.

## A call for engaged medical humanities

In this article, we have demonstrated how CoRe’s engaged approach to the medical humanities offers a compelling framework for conducting research that is at once academically rigorous and societally relevant. By proposing this framework, we have shown that while our engagement with both theory and practice can be conflicting and cumbersome, it is also a highly rewarding research approach, as it allows empirically informed theoretical contributions to various research fields, as well as theoretically based practice interventions addressing societal needs. Our take on the medical humanities is deeply rooted in the critical medical humanities’ call for entanglements in “real-world” settings (Fitzgerald and Callard 2016; Kristeva et al. 2018). On this basis, we suggest advancing the critical medical humanities by empirically exploring and stress-testing the entanglements in various real-world settings. In doing so, we have suggested a framework for the engaged medical humanities. More specifically, we have identified the specific kinds of troubles that arise when following our approach concerning project collaborations and knowledge production. These are not troubles that we wish to avoid; rather, as Haraway (2016) has put it, the aim is “staying with the trouble[s],” as they are highly important. Yet, it is for such troubles that we have gradually developed ad hoc handling strategies, as we have shown in the two cases discussed here. Furthermore, as part of our framework, we propose two sets of concepts through which to critically understand the engagements in both practical settings and theoretical discussions. First, we have suggested exploring our practice-based engagement with societal partners in terms of *trading zones* and *accountability* to understand the complexities, power relations, and ad hoc situations occurring in project partnerships that cannot be planned for or agreed upon in the initial phases of projects. These concepts have illuminated some of the ethical and practical conflicts and dilemmas that we, as researchers, must navigate, emphasizing skilled sensitivity (Whyte 2009), ongoing negotiations, and expertise regarding affectual, interpersonal, and group dynamics.

Second, we have focused on knowledge production in the engaged medical humanities through *theorizing and instrumentation*. As we have argued, both theoretical and conceptual research contributions and practice-based instruments and interventions are integral to our engaged medical humanities framework. In our analysis, we have shown how our diverse knowledge productions are deeply entangled in and informed by one another. Altogether, CoRe’s engaged medical humanities framework allows for a deeper understanding of health, illness, and treatment as socio-material phenomena intricately linked with political, economic, and cultural dimensions. In this way, the engaged medical humanities offer substantial opportunities for impacting and enriching both theoretical and practical frameworks, with possible inspirations and takeaways for fellow researchers interested in this field of study.

However, our specific approach is conditioned by the Danish context. On one hand, the research conducted at CoRe is shaped by some of the major current challenges facing research in Denmark, including short-term projects, precarious employment conditions, and time-limited research periods. In the case of the medical humanities, there is also a lack of earmarked funding and educational programs. On the other hand, the Danish context provides ample opportunities to engage in medical humanities research. For instance, there is a significant Danish funding landscape that prioritizes practice-based research and interdisciplinary collaborations. Furthermore, working as a research community, our engaged research approach develops and thrives as it continuously gathers and exchanges project experiences and trading zone maneuvering skills. Our many diverse projects over the last decade have given us a large network of collaborative partners inside and outside academia, and we benefit from an ongoing exchange and transfer of research competences between us and government agencies and municipalities, as former students and research colleagues from CoRe are in high demand. Our combined networks and knowledge products have an impact in these settings, as people, recommendations, instruments, and reports circulate and lead to changes because of their competences and high applicability in practice. At the same time, we have experienced great interest from our theoretically focused peers precisely because of our empirical sensitivity and practice-oriented work. While the critical medical humanities calls for an entangled medical humanities, our proposal, based on the last ten years of research, is this framework for approaching the entanglements with empirical sensitivity, in order to investigate how they unfold and shape lived lives.

## Data Availability

Not applicable. All participating informants have been promised anonymity. It is impossible to anonymize the dataset completely.

## Endnotes

^1^ CoRe is a research collective affiliated with the ethnology section at the Saxo Institute at the University of Copenhagen. It consists of ethnologists, historians, anthropologists, literature scholars, public health scholars, etc., working qualitatively with a broad understanding of health at the intersection of social science and the humanities. The projects hosted at CoRe are often engaged in interdisciplinary and cross-sectoral research collaborations.

^2^ This project is funded by three sources: the Velux Foundation (veluxfonden.dk/en), which distributes funds generated partly through returns on assets and partly through a share of the annual profits generated by the private corporate group, VKR Holding A/S; the Foundations fund research in the humanities, humanistic praxis interventions, and initiatives related to vision issues; Sygeforsikring Danmark (sygeforsikring.dk/sundhedsdonationer), a member-owned insurance association funding health-promoting projects in Denmark through donating parts of its returns on investments; and the Faculty of Health and Medical Sciences at the University of Copenhagen (for more information, see odont.ku.dk/Forskning/sund-mund-hele-livet/).

^3^ The funding source of this project is the Velliv Association (vellivforeningen.dk/soeg-stoette/), a philanthropic association for customers of the pension and insurance company Velliv. Approximately 20% of the returns go to charitable activities that promote mental health in Denmark, including the funding of research projects. The aim is to benefit and improve conditions for the Association’s target group of small and medium-sized companies by supporting the development of new initiatives and tools (for more information, see core.ku.dk/forskning/seniorpraksis/).

## References

[CR1] Aabo, Marie Gorm, Kathrine Mølgaard, and Aske Juul Lassen. 2023. The worn-out syndrome: Uncertainties in late working life triggering retirement decisions. *PLOS **ONE* 18(3): e0282905. 10.1371/journal.pone.0282905 10.1371/journal.pone.0282905 PMC10057830 36989274

[CR2] Barkin, Shari, David Schlundt, and Padget Smith. 2013. Community-engaged research perspectives: Then and now. *Academic Pediatrics* 13(2): 93–97. 10.1016/j.acap.2012.12.006 10.1016/j.acap.2012.12.006 23498079

[CR3] Birkbak, Andreas, Morten Krogh Petersen, and Torben Elgaard Jensen. 2015. Critical proximity as a methodological move in techno-anthropology. Techné: Research in Philosophy and Technology 19(2), 266–290. 10.5840/techne201591138

[CR4] Brody, H. 2011. Defining the medical humanities: Three conceptions and three narratives. *Journal of Medical Humanities,* 32, 1–7. 10.1007/s10912-009-9094-4 10.1007/s10912-009-9094-4 19936898

[CR5] Campus Engage. 2022a. Engaged research framework: A how-to guide. Campus Engage. https://www.iua.ie/wp-content/uploads/2023/12/Guide-IUA-Engaged-Research-Framework-2022_V7-11.pdf

[CR6] Campus Engage. 2022b. Engaged Research Principles and Good Practices. Campus Engage. https://www.iua.ie/wp-content/uploads/2023/12/Guide-IUA-Engaged-Research-Principles-Good-Practices-2022-Update_V5-471-3.pdf

[CR7] Carusi, Annamaria. 2016. Modelling systems biomedicine: Intertwinement and the ‘real’. In *The Edinburgh companion to the critical medical humanities*, eds. Anna Whitehead, Angela Woods, Sarah Atkinson, Jane Macnaughton, and Jennifer Richards, 50–65. Edinburgh University Press. 10.3366/j.ctt1bgzddd.7

[CR8] Clance, Pauline Rose, and Suzanne Ament Imes. 1978. The imposter phenomenon in high achieving women: Dynamics and therapeutic intervention. Psychotherapy: Theory, Research and Practice 15(3), 241–247. 10.1037/h0086006

[CR9] Collins, Harry, Robert Evans, and Mike Gorman. 2007. Trading zones and interactional expertise. *Studies in History and Philosophy of Science Part A* 38(4): 657–666. 10.1016/j.shpsa.2007.09.003 10.1016/j.shpsa.2015.07.004 26568093

[CR10] Cunliffe, Ann, and Giuseppe Scaratti. 2017. Embedding impact in engaged research: Developing socially useful knowledge through dialogical sensemaking. *British Journal of Management* 28(1): 29–44. 10.1111/1467-8551.12204

[CR11] Delgado, Ana, Kamilla Lein Kjølberg, and Fern Wickson. 2011. Public engagement coming of age: From theory to practice in STS encounters with nanotechnology. *Public Understanding of Science* 20(6): 826–845. 10.1177/0963662510363054

[CR12] Dolezal, Luna, and Matthew Gibson. 2022. Beyond a trauma-informed approach and towards shame-sensitive practice. *Humanities and Social Sciences Communications* 9(214): 1–10. 10.1057/s41599-022-01227-z 10.1057/s41599-022-01227-z PMC7612965 35791341

[CR13] Downey, Gary Lee, and Teun Zuiderent-Jerak. 2021. *Making and**doing: Activating STS through knowledge expression and travel*. MIT Press. 10.7551/mitpress/12321.001.0001

[CR14] Elgaard Jensen, Torben. 2020. Engaging the data moment. *Encounters* 11 (1): 89–116. 10.7146/stse.v11i1.135273

[CR15] Engebretsen, Eivind, Gina Fraas Henrichsen, and John Ødemark. 2020. Towards a translational medical humanity: Introducing the cultural crossings of care. *Medical Humanities* 46(2): 120-126. 10.1136/medhum-2019-011751 10.1136/medhum-2019-011751 PMC7402465 32341131

[CR16] Fitzgerald, Des, and Felicity Callard. 2016. Entanglement in the medical humanities. In *The Edinburgh companion to the critical medical humanities*, eds. Anna Whitehead, Angela Woods, Sarah Atkinson, Jane Macnaughton, and Jennifer Richards, 35–49. Edinburgh University Press. 10.3366/j.ctt1bgzddd.6 27536763

[CR17] Folker, Louise, Astrid Pernille Jespersen, and Esben Boeskov Øzhayat. 2025. Tooth shame: An ethnographic study of the choreographies of tooth shame in Danish elderly care. *Social Science & Medicine, 320,* 115722. 10.1016/j.socscimed.2024.115722 10.1016/j.socscimed.2024.117500 39644775

[CR18] Galison, Peter. 1999. Trading zone: Coordinating action and belief (1998 abridgment). In *The science studies reader*, ed. Mario Biagioli, 137–160. London: Routledge.

[CR19] Haapanen, Krista A., and Brian D. Christens. 2021. Community-engaged research approaches: Multiple pathways to health equity. *American Journal of Community Psychology* 67(3–4): 331–337. 10.1002/ajcp.12529 10.1002/ajcp.12529 34312882

[CR20] Hansson, Kristofer, and Rachel Irwin, eds. 2020. Introduction: Movement of knowledge. In Movement of knowledge: Medical humanities perspectives on medicine, science, and experience. Nordic Academic Press. 10.21525/kriterium.24

[CR21] Haraway, Donna J. 2003. *Companion**species manifesto. Dogs, people, and significant otherness.* Chicago: Prickly Paradigm Press.

[CR22] Haraway, Donna J. 2016. *Staying with the trouble: Making kin in the Chthulucene*. Durham, NC: Duke University Press. 10.1215/9780822373780

[CR23] Heney, Veronica, and Branwyn Poleykett. 2022. The impossibility of engaged research: Complicity and accountability between researchers, ‘publics’ and institutions. *Sociology of Health and Illness* 44(S1): 179–194. 10.1111/1467-9566.13418 10.1111/1467-9566.13418 PMC7617641 34874575

[CR24] Hinchcliffe, Stephen, Mark A. Jackson, Katrina Wyatt, Anne E. Barlow, Manuela Barreto, Linda Clare, Michael H. Depledge, Robin Durie, Lora E. Fleming, Nick Groom, Karyn Morrissey, Laura Salisbury, and Felicity Thomas. 2018. Healthy publics: Enabling cultures and environments for health. *Palgrave Communications* 4(1): 1–10. 10.1057/s41599-018-0113-9 10.1057/s41599-018-0113-9 PMC5978671 29862036

[CR25] Jespersen, Astrid Pernille. 2021. Det sunde liv og/eller det gode liv? *Culture and History: Student Research Papers* 5(02): 66–73. 10.7146/chku.v5i02.127338

[CR26] Klotz, Anna-Luisa, Alexander Jochen Hassel, Johannes Schröder, Peter Rammelsberg, and Andreas Zenthöfer. 2018. Is compromised oral health associated with a greater risk of mortality among nursing home residents? A controlled clinical study. *Aging Clinical and Experimental Research* 30(6): 581–588. 10.1007/s40520-017-0811-y 10.1007/s40520-017-0811-y 28856592

[CR27] Kristeva, Julia, Marie Rose Moro, John Ødemark, and Eivind Engebretsen. 2018. Cultural crossings of care: An appeal to the medical humanities. *Medical Humanities* 44(1): 55–58. 10.1136/medhum-2017-011263 10.1136/medhum-2017-011263 PMC5869466 28935631

[CR28] Lloyd, Michener, Jennifer Cook, Syed M. Ahmed, Michael A. Yonas, Tamera Coyne-Beasley, and Sergio Aguilar-Gaxiola. 2012. Aligning the goals of community-engaged research: Why and how academic health centers can successfully engage with communities to improve health. *Academic Medicine* 87(3): 285–291. 10.1097/ACM.0b013e3182441680 10.1097/ACM.0b013e3182441680 PMC3292771 22373619

[CR29] Macnaughton, Jane. 2011. Medical humanities’ challenge to medicine. *Journal of Evaluation in Clinical Practice* 17(5): 927–932. 10.1111/j.1365-2753.2011.01728.x 10.1111/j.1365-2753.2011.01728.x PMC4439737 21851510

[CR30] Mikesell, Lisa, Elisabeth Bromley, and Dimitri Khodyakov. 2013. Ethical community-engaged research: A literature review. *American Journal of Public Health* 103(12): e7–e14. 10.2105/AJPH.2013.301605 10.2105/AJPH.2013.301605 PMC3828990 24134352

[CR31] Oladi, Soudeh. 2013. The instrumentalization of education. The Atlantic Journal of Graduate Studies in Education: Special Ed: 1–15. https://www.academia.edu/9494191/The_Instrumentalization_of_Education.

[CR32] Paphitis, Sharli Anne. 2017. The possibility of addressing epistemic injustice through engaged research practice: Reflections on a menstruation-related critical health education project in South Africa. *Critical Public Health* 28(3): 363–372. 10.1080/09581596.2017.1418500

[CR33] Rouxel, Patrick, Anja Heilmann, Panayotes Demakakos, Jun Aida, Georgios Tsakos, and Richard G. Watt. 2017. Oral health-related quality of life and loneliness among older adults. *European Journal of Ageing* 14(2): 101–109. 10.1007/s10433-016-0392-1 10.1007/s10433-016-0392-1 PMC5435788 28579932

[CR34] Santos, J.M., Horta, H. & Luna, H. 2022. The relationship between academics’ strategic research agendas and their preferences for basic research, applied research, or experimental development. *Scientometrics* 127, 4191–4225. 10.1007/s11192-022-04431-5 10.1007/s11192-022-04431-5 PMC9285191 35855468

[CR35] Stengers, Isabelle. 2005. The cosmopolitical proposal. In *Making things public: Atmospheres of democracy*, eds. Bruno Latour and Peter Weibel, 994–1003. Cambridge, MA: MIT Press.

[CR36] Viney, William, Felicity Callard, and Angela Woods. 2015. Critical medical humanities: Embracing entanglement, taking risks. *Medical Humanities* 41(1): 2–7. 10.1136/medhum-2015-010692 10.1136/medhum-2015-010692 PMC4484495 26052111

[CR37] Whyte, Susan Reynolds. 2009. Health identities and subjectivities. *Medical Anthropology Quarterly* 23: 6–15. 10.1111/j.1548-1387.2009.01034.x 10.1111/j.1548-1387.2009.01034.x 19449709

[CR38] Wulff, Anna, and Aske Juul Lassen. 2024. Capacity for competence development: Unlocking potential for lifelong learning in later working life. *Journal of Aging and Social Policy*: 1–22. 10.1080/08959420.2024.2349492 10.1080/08959420.2024.2349492 38739387

